# Maintenance of arterial catheters with heparin: should we continue?

**DOI:** 10.1186/cc9498

**Published:** 2011-03-11

**Authors:** N Catorze, S Teixeira, J Cabrita, J Carreto, V Vieira, S Gonçalves, A Frade, J Martins

**Affiliations:** 1 Centro Hospitalar Médio Tejo, Abrantes, Portugal

## Introduction

In ICU settings arterial catheters (AC) are used to manage critically ill patients. Maintaining the patency of these catheters is important for continuous hemodynamic evaluation and therapeutic adjustment. Heparinized solutions are used for this purpose although the increasing literature describes the use of saline solutions for the same reason. The authors compare the use of heparinized versus saline solution in the maintenance of ACs and to detect changes in aPTT, platelet count, and local inflammatory signs, in a double-blind randomized trial.

## Methods

During 80 days all ICU patients with ACs were randomized to receive heparinized solution (5 IU/ml) or saline solution. AC patency and functionality was compared in both every 6 hours, and aPTT, platelet count and local inflammatory signs each 24 hours. Patients with thrombocytopenia, receiving anticoagulant or fibrinolytic treatment were excluded.

## Results

Two hundred days of ACs were observed in 49 patients, during which 110 days were with saline solutions and the rest were heparinized. Seven patients were excluded. The median duration of catheters in place was 4.4 days in the saline group and 3.8 days in the heparinized group. We recorded two ACs with local inflammatory signs in the heparinized group that were replaced in a septic context. One local hemorrhage and one AC obstruction were observed in the heparinized group versus no hemorrhage and three AC obstructions in the saline group. No other differences were obtained.

## Conclusions

The generalized use of heparin solution for AC maintenance does not seem to be adequate. In this study the comparison of the two populations revealed the same results despite the solution used. These results do not encourage the use of heparinized solutions because they do not have an effective cost/benefit relation and due to the potential iatrogenic problems described in the literature.
